# Norovirus Persistence in Oysters to Prolonged Commercial Purification

**DOI:** 10.3390/pathogens10080944

**Published:** 2021-07-28

**Authors:** Roberta Battistini, Chiara Masotti, Valeria Listorti, Elisabetta Suffredini, Cristiana Maurella, Aitor Garcia-Vozmediano, Erica Costa, Francesco Iacona, Mino Orlandi, Carlo Ercolini, Laura Serracca

**Affiliations:** 1 Istituto Zooprofilattico Sperimentale del Piemonte, Liguria e Valle d’Aosta, 10154 Turin, Italy; chiara.masotti@izsto.it (C.M.); valeria.listorti@izsto.it (V.L.); cristiana.maurella@izsto.it (C.M.); aitor.garciavozmediano@izsto.it (A.G.-V.); carlo.ercolini@izsto.it (C.E.); laura.serracca@izsto.it (L.S.); 2 Department of Food Safety, Nutrition and Veterinary Public Health, Istituto Superiore di Sanità, 00161 Rome, Italy; elisabetta.suffredini@iss.it; 3 Liguria Local Health Unit-ASL 5, Complex Unit of Hygiene of Foods and Animal Origin, 19122 La Spezia, Italy; Erica.costa@asl5.liguria.it (E.C.); Francesco.iacona@asl5.liguria.it (F.I.); mino.orlandi@asl5.liguria.it (M.O.)

**Keywords:** *Crassostrea gigas*, depuration, norovirus, food safety, real-time qPCR

## Abstract

Depuration is generally the main treatment employed for bivalve mollusks harvested from contaminated sites. Commercial depuration has demonstrated to be effective for removal of bacterial pathogens, although it probably provides only limited efficacy against human enteric viruses. We evaluated the quantitative reduction of norovirus (NoV) genogroups I and II in naturally contaminated oysters after 1, 4, and 9 days of depuration. The process was conducted in an authorized depuration plant, and NoV concentration was determined by RT-qPCR according to ISO 15216-1:2017 method. Regardless of the NoV genogroup, our results showed no significant reduction in NoV concentration after 1 day of depuration. Higher mean reduction (68%) was obtained after 4 days of treatment, while no further increase was observed after 9 days. Overall, reduction was highly variable, and none of the trials showed statistically significant reduction in NoV RNA concentration at the end of each depuration period. Indeed, NoV concentration remained high in 70% of samples even after 9 days of depuration, with values ranging between 4.0 × 10^2^ and 2.3 × 10^4^ g.c./g. These results indicate that an extension of commercial depuration time does not appear to be effective for reducing or eliminating NoV in oysters.

## 1. Introduction

Oysters are a well-documented source of norovirus (NoV) infection as bivalve shellfish can accumulate viral particles by filtration of contaminated water, and oysters pose a particular risk to human health since they are routinely consumed raw. In 2019, NoV was the second most frequently reported causative agent in foodborne outbreaks in the EU, being associated with 457 human outbreaks and 11,125 related illnesses (22.5% of total cases), mostly for consumption of fish and fishery products [1]. NoVs are nonenveloped viruses belonging to the *Caliciviridae* family, with a single-stranded positive-sense RNA genome. They are classified into seven genogroups (GI to GVII) and 38 genotypes. Genogroups I, II, and IV infect humans [2]. The infection is self-limiting in healthy adults, and clinical symptoms tend to last for 2–3 days; however, the clinical picture may be more severe and last longer in young children, elderly people, and individuals who have impaired immune functions, requiring hospitalization [3]. Additionally, NoV also has a major economic impact in developed countries and particularly in shellfish industry [4]. Shellfish production areas in Europe are classified into three categories (A, B, or C) on the basis of the presence and levels of the fecal indicator *E. coli* [5]. Shellfish postharvest treatments are prescribed depending on the classification. In Europe, around 60% of the total oyster production comes from class B areas [6]. To ensure consumers’ health protection, before commercialization, shellfish harvested from class B areas must be subjected to purification in an approved plant, to relaying in approved class A relaying areas, or to an EU-approved heat treatment process. Depuration is a postharvest treatment that involves placement of shellfish in tanks of clean seawater usually pretreated with UV light, chlorine, iodophors, or ozone to reduce contaminant levels [7]. During depuration, bivalve mollusks eliminate pathogens and contaminants present in the digestive tract [8]. A wide variety of depuration periods are used around the world, varying from a few hours to several days. The industry targets the period primarily at the removal of fecal indicator bacteria *E. coli* when a minimum period is not specified by the competent authority. Complete elimination of *E. coli* normally occurs well within 48h, although it is also recognized that some species of bivalves may require more or less time. Often, shorter periods are used in some countries, for example, depuration periods of 18–24 h are commonly used in Italy. Reducing the viral contamination in shellfish is essential for preserving public health. However, virus removal is known to be less effective than bacterial removal, and the compliance with standards cannot guarantee the viral absence, as shown by cases where oysters compliant to the legislative microbiological standards for *E. coli* were associated with NoV outbreaks [2,9]. Therefore, as many studies confirm, depuration as currently performed appears ineffective in guaranteeing oysters free from viral pathogens [10,11]. Parameters of seawater, such as temperature, salinity, dissolve oxygen content, turbidity, and phytoplankton concentration, can affect shellfish filtration efficiency, playing a relevant role in the effectiveness of depuration processes [12,13,14,15]. Some studies suggest that, in the same condition, the effectiveness of depuration in mollusks might differ between bivalve species and virus types/strains [10,16,17,18,19,20]. Specific NoV ligands found in oyster tissues, for example, may contribute to the persistence of viral particles and resistance to depuration [10,21,22,23]. Based on EFSA recommendations, depuration and relaying may be improved by optimizing process parameters to enhance NoV reduction (e.g., depuration times). Since limited data are currently available, further studies are needed to establish and optimize the effectiveness of depuration and relaying for NoV reduction using the standardized CEN method [24]. To this aim, in this study, we indagate the effect of an increase in purification times on NoV reduction in oysters. Most of the studies regarding the purification rate of noroviruses from oysters have been done with artificially contaminated oysters [11,13,19,25,26]. As far as we know, only two research studies have evaluated the effectiveness of depuration process in the reduction of NoV in naturally contaminated oysters (species *Crassostrea gigas*) subjected to purification for 5 [18] and 7 days [27]. Therefore, in our investigation, we chose to indagate NoV reduction in naturally contaminated oysters subjected to purification processes of different lengths (1, 4, and 9 days postharvest) in a commercially authorized plant. The purification time of 1 day was chosen because it is that routinely used in the plant studied, 4 days is an interesting time as it seems able to significantly reduce *V. parahaemolyticus*, an other important pathogen present in oysters [28], and finally, 9 days is not yet sufficiently investigated. The data provided in this study will help evaluate the effectiveness of postharvest treatments of oysters with high NoV loads in real commercial purification plants.

## 2. Results

**Figure 1:**
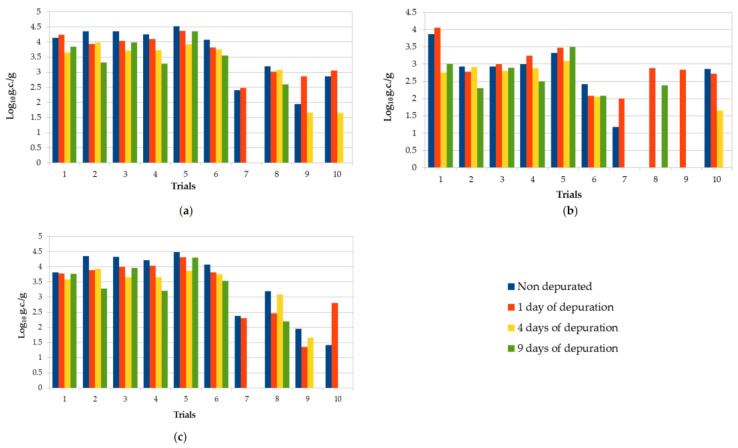
Comparison of NoV RNA concentration found in all trials of oysters subjected to purification processes before and after different depuration times (1, 4, and 9 days postharvest): (**a**) NoV GI + GII; (**b**) NoV GI; (**c**) NoV GII. Values are expressed in log_10_ genome copies/gram.

Table 1 and Figure 1 show the NoV RNA values found in oysters before and after the three purification periods. Data expressed in genomic copies per gram (g.c./g) are reported separately for NoV genogroup I and II and for the sum of the two genogroups. In total, we examined 10 sets of oyster samples (nondepurated, 1, 4, and 9 days of depuration).

Physiochemical parameters of seawater during oysters’ depuration periods are summarized in Table 2.

The depuration plant is located near the sea, taking the water from the environment. This implies variable parameters along the year. Temperature, in particular, ranged from 12 to 18.2 °C depending on the period. However, within a single purification trial, the variation was never greater than 3 °C. The other parameters showed no significant variations within the same trial. All nondepurated samples analyzed were positive for at least one NoV genogroup and 8/10 were positive for both of them. The concentration of NoV GI + GII ranged from 7.5 × 10^2^ g.c./g to 3.3 × 10^4^ g.c./g. Only in one sample, NoV concentration was <LOQ. NoV GII was the most prevalent genogroup in nondepurated samples, and 80% of the samples showed higher viral levels compared with NoV GI (Table 1). No significant differences of viral concentrations were observed after 1 day of depuration in both genogroups (*p* > 0.05); in particular, six samples showed higher concentrations of NoV GI after 1 day of purification compared with untreated samples. The highest mean reduction of NoV (68 %) was generally obtained after 4 days of depuration, followed by no further relevant decrease after 9 days. Viral reduction was greater for genogroup II compared with genogroup I: on average, in the ten trials, after 9 days of depuration, 62.3% (−0.42 log) for GII vs. 55.7% (−0.36 log) for GI. However, in some samples, an increase in virus concentration was observed after depuration compared to nondepurated samples. Overall, the reduction of NoV RNA was very variable, and none of the trials showed a significant reduction (*p* > 0.05) in NoV RNA concentrations at the end of each depuration period. Seven out of ten contaminated samples were still positive after the ninth day of depuration, with NoV concentrations ranging between 4.0 × 10^2^ and 2.3 × 10^4^ g.c./g.

## 3. Discussion

Few studies investigated reduction of NoV concentration in naturally contaminated oysters in nonexperimental depuration settings. To our knowledge, this study investigated for the first time the effectiveness of commercial depuration in an authorized plant for the reduction of NoV in naturally contaminated oysters of the species *Crassostrea gigas*, extending the purification time up to 9 days. Our results showed that commercial purification does not significantly reduce NoV from oysters, even if it is prolonged up to 9 days. Otherwise, Younger et al., 2020, after 5 days of depuration at 18 °C, found 60% removal of NoVGII and 16% of NoV GI, while Rupnik et al., 2021 found, after 7 days at 12–16 °C, a reduction of 67.58% for NoVGI and 83.95% for NoVGII. However, Rupnik found a concentration of NoV in oyster lower than that found in this study, and showed that in commercial settings, the ability to reduce the NoV concentration in oysters to values <LOQ differed when contaminated with a concentration below and above 1000 c.g./g. In fact, samples contaminated with norovirus GII at concentrations >1000 c.g./g were reduced to concentrations <LOQ in a lower percentage (40%) than the samples contaminated with <1000 norovirus GII c.g./g (78.6%) were. In our study, the NoV GII concentrations were >1000 g.c./g in 70% of samples, which may explain the different results obtained in the two studies. Overall, the variability between the different trials was significant and does indicate that not all depuration runs obtained similar reductions. This variability could be caused by several factors; in particular, the differences in the physiological activity of shellfish, but also the different environmental seawater condition between samplings. Other studies have shown that is a large variability in virus uptake between individual shellfish [19]; moreover, each sample is obtained from a pool of 10 individuals, so each animal may have accumulated norovirus in a different way and respond differently to stress factors and consequently, to depuration condition. All these factors may have contributed to the variability in the results. The data obtained in our trials showed a higher persistence of NoV GI during the purification process compared with NoV GII, highlighting a different behavior of the two NoV genogroups in oysters, as already evidenced in other studies. Many authors, indeed, suggested that oysters are not a passive filter but NoV genogroups bind to different ligands inside the oyster tissues [23,29]. In particular, NoV GI binds to the midgut and digestive diverticula but not to gills or mantle, whereas NoV GII binds to all of these tissues [10,30]. These specific ligand interactions could explain the viral persistence in oysters despite the depuration and the different behavior of the two genogroups. Furthermore, in the majority of trials carried on in our study, we observed, especially for NoV GII, a steeper decrease of NoV concentration after 4 days of depuration, followed by a stabilization at 9 days. These results are consistent with both laboratory-based depuration studies and commercial-based depuration studies, which evidenced a two-phase depuration kinetics in many mollusk species, although with different timing in the different species [10,16,31], probably due to the variability among species and the capability of NoV to resist to suboptimal conditions during depuration process, as suggested by other authors [14,16]. In several studies on oysters, indeed, a maximum viral reduction was obtained in the first 3–4 days of depuration, while no further significative reduction was observed by extending the time [26,32]. Some authors suggest that the more rapid viral reduction in the first phase could be related to extracellular digestion and purging of the digestive tract, while in the second phase, viral reduction is more difficult and slower because NoV is specifically attached to ligands present on oysters’ gastrointestinal cells or in other parts outside the digestive tract lumen, like hemocytes [17,20,23,33,34]. Le Guyader et al. (2006) showed that there is a carbohydrate on the surface of the digestive tissues of oysters (*Crassostrea gigas*) that can specifically bind norovirus particles [23]. This binding site has been mapped in the virus in a restricted area of the viral capsid P2 domain, involved in receptor binding and immunogenicity [35]. Mutations in key residues in this domain inactivate the binding activity to histoblood group structures [23]. Therefore, capsid integrity is necessary for viable viruses to bind to the oyster’s ligand. It follows that the detection of viral RNA in the second-phase depuration kinetics could indicate the presence of infectious viruses, because the integrity of the capsid is fundamental also to the integrity of viral RNA. The RNA genome of NoV indeed is normally protected by a resilient capsid, but damage to the capsid’s integrity makes the RNA molecule highly susceptible to degradation by ubiquitous RNases in the environment. The extension of the purification period could serve as an indicator of the possible presence of infectious noroviruses in the absence of a practical culture technique for routine analysis and could be taken into consideration to assess the risk associated with the consumption of bivalve mollusks.

## 4. Materials and Methods

### 4.1. Sampling

The study was carried out using samples of naturally contaminated oysters (*Crassostrea gigas*) collected from a class B shellfish farming area [5] in the northwest of Italy. A minimum of forty oysters were collected from a single sampling point every 2 weeks, for a total of 10 samplings, during the coldest months of the year (from December to April) when the viral load is supposed to be highest. From each sampling, a depuration trial was performed: 10 oysters were tested for both NoV GI and GII before depuration (T0), while the other 30 oysters were transferred to an authorized commercial depuration plant and analyzed for NoV GI and GII after 1, 4, and 9 days of purification treatment (10 individuals analyzed for each depuration time).

### 4.2. Depuration System

The depuration plant consisted of a vertical system of isothermal containers (bins) with a volume of 600 L. Bins were filled with seawater pumped with a flow rate of about 4 × 10^3^ L/h. This seawater was purified through quartz sand filters, biological filters, and a skimmer to remove dissolved macromolecules. It was subsequently purified using both an Ozone sterilizer an UVC apparatus, the latter equipped with 4 lamps that deliver a dose of 39 mJ/cm^2^ per each passage. Seawater parameters of temperature, pH, salinity, and O_2_ were controlled during the depuration period and recorded at each sampling.

### 4.3. NoV RNA Extraction

Viral recovery from oysters was carried out as reported in ISO 15216-1:2017 method [36]. Briefly, digestive tissue was removed by dissection from each oyster, pooled, and homogenized. Aliquots of 2 g were spiked with 10 μL of process control virus (Mengovirus), digested with 2 mL of proteinase K (0.1 mg/mL) at 37 °C for 60 min with shaking at 350 rpm, and then maintained at 60 °C for 15 min to inactivate the enzyme. Then, samples were centrifuged at 3000× *g* for 5 min, supernatants were collected, and volumes recorded. Viral RNA was extracted using NucliSENS^®^ magnetic extraction reagents (BioMerieux, Marcy l’Etoile, France) according to the manufacturer’s instructions from 500 μL of the supernatants. Finally, RNA was eluted (100 μL) and used immediately for real-time RT-PCR analysis or stored at −80 °C.

### 4.4. Norovirus Quantification

NoV RNA quantification was performed by real-time RT-PCR using primers, probes, and amplification conditions reported in ISO 15216-1:2017. Reactions were carried out using the Biorad CFX96TM Real-Time PCR thermocycler (Biorad, Hercules, CA, USA) and the RNA Ultrasense One-Step qRT-PCR system (Invitrogen, Carlsbad, CA, USA). All samples were analyzed for NoV genogroup I (GI) and genogroup II (GII). Genome quantification was estimated by comparing the sample *C*q value to five-point standard curves (one for each target) constructed with serial dilution of dsDNA standard for NoV GI and GII, sup-plied by the Italian National Reference Laboratory (NRL) for Foodborne Viruses. Results were expressed as genome copies per gram (g.c./g) and were calculated based on the volume of extract analyzed. The limit of quantification (LOQ) was 140 g.c./g for NoV GI and 130 g.c./g for NoV GII. In accordance with ISO 15216-1:2017, extraction efficiency was assessed through the recovery of the process control (Mengovirus) by comparing the *C*q value of Mengovirus obtained in spiked samples with that extracted by viral stock. Results with extraction efficiency greater than 1% were considered acceptable. In addition, to test the presence of RT-PCR inhibitors, 1 µL of external control RNA for both GI and GII was added to samples. The *C*q value obtained in samples spiked with external control RNA was then compared to that obtained in samples without external control and used to evaluate inhibition. Values with RT-PCR inhibition ≤75% were considered valid.

### 4.5. Statistical Analysis

Statistical analyses were performed using STATA 16.1 version for Windows. The nonparametric Kruskal–Wallis rank sum test was performed to evaluate changes in the NoV concentration (genomic copies/gram) uncovered in the pool of oysters (*n* = 40) at different times of depuration and by considering both the single occurrence of genogroups NoV GI and GII and their combination (GI + GII) within the same pool. A two-tailed significance level of α = 0.05 was adopted.

## 5. Conclusions

The results of this study seem to indicate that an extension of commercial depuration time up to 9 days cannot reduce significantly or eliminate NoV in oysters. After 1, 4, and 9 days of purification, 100%, 90%, and 70% of the samples, respectively, were still positive for at least one NoV genogroup. Our trials showed better removal of NoV genogroup II compared to genogroup I and a highest mean reduction of NoV after 4 days (68%). However, the high variability of the data obtained in the trials did not allow to obtain a statistically significant reduction. Therefore, in order to increase consumers’ food safety and given the possible introduction of new criteria for NoV in oysters in the near future, the investigation of new solutions for the reduction of NoV contamination in oysters remains a priority.

## Figures and Tables

**Table 1:** Quantification of Norovirus (NoV) concentrations, expressed as genome copies/gram, in 10 sampled trials of *Crassostrea gigas* before and after different depuration times (from 1 day to up to 9 days postharvest). Note: LOQ is 140 g.c./g for NoV GI and 130 g.c./g for NoV GII; n.d.: not detected.

	Trials	Nondepurated (g.c/g)	1 Day Depuration (g.c./g)	4 Days Depuration (g.c./g)	9 Days Depuration (g.c./g)
**NoV** **GI + GII**	1	1.4 × 10^4^	1.8 × 10^4^	4.5 × 10^3^	6.9 × 10^3^
	2	2.3 × 10^4^	8.4 × 10^3^	9.5 × 10^3^	2.1 × 10^3^
	3	2.2 × 10^4^	1.1 × 10^4^	5.2 × 10^3^	9.9 × 10^3^
	4	1.8 × 10^4^	1.3 × 10^4^	5.4 × 10^3^	1.9 × 10^3^
	5	3.3 × 10^4^	2.4 × 10^4^	8.4 × 10^3^	2.3 × 10^4^
	6	1.2 × 10^4^	6.7 × 10^3^	5.7 × 10^3^	3.5 × 10^3^
	7	2.6 × 10^2^	3.1 × 10^2^	n.d.	n.d.
	8	1.6 × 10^3^	1.1 × 10^3^	1.2 × 10^3^	4.0 × 10^2^
	9	<LOQ	7.2 × 10^2^	4.6 × 10^1^	n.d.
	10	7.5 × 10^2^	1.2 × 10^3^	4.5 × 10^1^	n.d.
**NoV GI**	1	7.4 × 10^3^	1.1 × 10^4^	5.8 × 10^2^	1.0 × 10^3^
	2	8.5 × 10^2^	6.1 × 10^2^	8.4 × 10^2^	2.0 × 10^2^
	3	8.6 × 10^2^	1.0 × 10^3^	6.3 × 10^2^	8.0 × 10^2^
	4	9.9 × 10^2^	1.7 × 10^3^	7.6 × 10^2^	3.2 × 10^2^
	5	2.1 × 10^3^	2.9 × 10^3^	1.2 × 10^3^	3.1 × 10^3^
	6	2.6 × 10^2^	<LOQ	<LOQ	<LOQ
	7	<LOQ	<LOQ	n.d.	n.d.
	8	n.d.	7.6 × 10^2^	n.d.	2.4 × 10^2^
	9	n.d.	7.0 × 10^2^	n.d.	n.d.
	10	7.2 × 10^2^	5.2 × 10^2^	<LOQ	n.d.
**NoV GII**	1	6.6 × 10^3^	6.1 × 10^3^	3.9 × 10^3^	5.9 × 10^3^
	2	2.2 × 10^4^	7.8 × 10^3^	8.7 × 10^3^	1.9 × 10^3^
	3	2.1 × 10^4^	1.0 × 10^4^	4.6 × 10^3^	9.2 × 10^3^
	4	1.7 × 10^4^	1.1 × 10^4^	4.6 × 10^3^	1.6 × 10^3^
	5	3.1 × 10^4^	2.1 × 10^4^	7.2 × 10^3^	2.0 × 10^4^
	6	1.2 × 10^4^	6.6 × 10^3^	5.6 × 10^3^	3.4 × 10^3^
	7	2.4 × 10^2^	2.0 × 10^2^	n.d.	n.d.
	8	1.6 × 10^3^	2.9 × 10^2^	1.2 × 10^3^	1.6 × 10^2^
	9	<LOQ	<LOQ	<LOQ	n.d.
	10	<LOQ	6.4 × 10^2^	n.d.	n.d.

**Table 2:** Physiochemical parameters of seawater measured during oysters’ depuration period. Data are reported as minimum and maximum value for each parameter during each trial.

Trials	T (°C)	pH	Salinity (‰)	Dissolved O_2_ (%)
**1**	12–15	7.9	38	95–96
**2**	12.5–14.5	7.9	38	95–96
**3**	12–13.5	7.9	38	96
**4**	14–15	7.8–7.9	38	92–96
**5**	13–14.7	7.9–8	38	100–105
**6**	15.2–16.9	7.9	38	93–94
**7**	16.2–17	7.2–7.5	38	75
**8**	16.7–18.2	7.2–7.8	38	75–93
**9**	12–15	6.8–7.8	38	75
**10**	14–16.9	6.8–7.5	38	75
